# The Reformed Public House

**Published:** 1924-06

**Authors:** A. W. Simons


### CORRESPONDENCE.

**[Correspondence on all subjects is invited, but we cannot in any way be responsible for the opinions expressed by our correspondents. No communication can be entertained if the name and address of the correspondent are not given as a guarantee of good faith, but not necessarily for publication. All correspondents should write on one side of the paper only, and we cannot find space for very long letters.]**

### The Reformed Public House.

*To the Editor of* The Hospital and Health Review.

Sir,—I am sorry I misrepresented the views of Mr. Dering. It appears now that he does not belong to that school of thought that would nationalise everything, but advocates only the nationalisation of the beverage trade.

Mr. Dering's chief complaint is that the trade have failed to keep their part of the bargain which requires licensees to provide a real public house in its widest sense; yet when attempts are made to induce licence holders to keep to their part of the bargain, as would be the case by the passage of a Bill on the lines of the Public House Improvement Bill, he and a host of other correspondents of the Temperance Legislation League rush into print with letters denouncing the Bill.

Mr. Dering is a staunch supporter of the Carlisle system, where public houses have been more or less reformed. Will he permit me to assume that these reforms have increased the facilities for doing more trade in beer, as he alleges the Bill above referred to is designed for? If so, how can he hold up the Carlisle experiment as an example of what the State can do in the matter of promoting sobriety?

A. W. Simons.

